# The high cost of movement in an arid working landscape for an endangered amphibian

**DOI:** 10.1002/ece3.11259

**Published:** 2024-04-16

**Authors:** Colin W. Brocka, Maria Vittoria Mazzamuto, John L. Koprowski

**Affiliations:** ^1^ School of Natural Resources and the Environment University of Arizona Tucson Arizona USA; ^2^ Haub School of Environment and Natural Resources University of Wyoming Laramie Wyoming USA

**Keywords:** *Ambystoma mavortium stebbinsi*, Arizona, connectivity, desert, endangered species, survival

## Abstract

Connectivity is essential for the maintenance of genetic diversity and stability of wildlife populations. Drought and changing precipitation regimes have caused natural aquatic amphibian breeding habitats to disappear or become isolated and have led to the replacement of natural surface water with artificial livestock water tanks. Terrestrial movement is the only means of responding to aquatic threats in arid landscapes and to allow population connectivity. Aridity may present an impenetrable barrier in hydrologically fragmented environments. We used a facultatively paedomorphic and federally endangered salamander to assess the challenges of movement across arid working lands. Sonoran tiger salamanders (*Ambystoma mavortium stebbinsi*) are endemic to the San Rafael Valley of southeastern Arizona, United States of America, where they depend on livestock water tanks as breeding habitat. The ecology of this species' metamorphs outside of stock tanks is virtually unknown. To assess survival on the landscape during terrestrial movement we used radio‐transmitters to track 78 adult metamorphosed salamanders over 2 years. Sonoran tiger salamanders moved up to 1 km from the tank edge, and average distances moved of over 400 m were higher than most *Ambystoma* species. However, during the study period, none reached neighboring stock tanks. We found high mortality due to predation and desiccation. Individuals that dispersed to terrestrial habitat in summer survived longer than individuals that dispersed in spring. High mortality suggests terrestrial movement is exceptionally risky and may contribute to isolated subpopulations and elevated levels of inbreeding. Conservation actions that improve and maintain artificial aquatic habitats as well as increase connectivity may improve long‐term management for pond‐breeding amphibians in arid regions.

## Graphical abstract

Dispersal is the only means of responding to aquatic threats in arid landscapes and thus is critical for maintenance of population dynamics. We studied the movement ecology and survival upon terrestrial movement of the federally endangered Sonoran tiger salamanders (*Ambystoma mavortium stebbinsi*) endemic to the southeast Arizona, USA. High mortality suggests terrestrial movement is exceptionally risky and may contribute to isolated subpopulations and elevated levels of inbreeding caused by rare reaching of neighboring stock tanks.

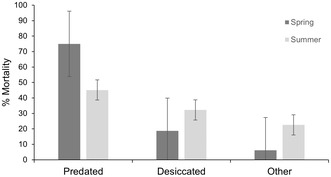

## 1 INTRODUCTION

Connectivity is essential for the maintenance of genetic diversity and the stability of wildlife populations by facilitating interactions between subpopulations (Goodwin & Fahrig, 2002; Hanski, 1998). Insufficient connectivity can lead to isolated populations that are more vulnerable to demographic stochasticity or extreme environmental events (O'Grady et al., 2006; Semlitsch & Bodie, 1998). In addition to the fragmentation caused by human land use, climate change has led to the increased occurrence of extreme weather events, such as drought, that can change hydrologic regimes and reduce connectivity of amphibian habitats (Becker et al., 2007; Mims et al., 2015; Semlitsch, 2000; Sodhi et al., 2008). This makes large‐bodied amphibians with restricted ranges particularly vulnerable to being added the IUCN Red List (Sodhi et al., 2008) and important and excellent models for studies about the costs of terrestrial movement.

From 1980 to 2004, habitat loss, weather extremes, and restricted ranges increased the risk of decline of nearly 2500 amphibians worldwide (Sodhi et al., 2008). This is especially true in the arid southwestern United States, where drought and changing precipitation regimes have caused natural aquatic amphibian breeding habitats to become highly isolated or disappear completely (Cook et al., 2015; Marshall et al., 2010). Areas whose streams prior to 1880 coursed unimpeded across alluvial fills in shallow, braided channels, often through lush marshes, have been reduced in size, artificially maintained, or variously modified with stabilization of flow by upstream dams, channelization, and desiccation by diversion and pumpage (Hendrickson & Minckley, 1985). This has resulted in a great reduction of natural stream communities in the desert of the Southwest (Hendrickson & Minckley, 1985) and has led to the construction in the landscape of earthen impoundments (hereafter, stock tanks) for water supply in locations that altered water distribution to align the distribution of free‐ranging livestock and forage (Nichols & Degginger, 2021). With the continuing disappearance of the natural stream communities, stock tanks have now become important for biodiversity of arid regions by providing additional aquatic habitat and stepping stones for connectivity (Brainwood & Burgin, 2009), despite often poor water quality (McIntyre et al., 2016; Schmutzer et al., 2008). These stock tanks, which can either be seasonal or perennial depending on drought conditions, serve as some of the only breeding habitat for many pond‐breeding amphibians such as endangered Sonoran tiger salamanders (*Ambystoma mavortium stebbinsi*).

Sonoran tiger salamanders are endemic to the San Rafael Valley and surrounding mountain foothills of southeastern Arizona and adjacent Sonora, Mexico, which are characterized by winter rains from December through February and heavy summer monsoon rains from late June to August that provide much of the annual rain total (Adams & Comrie, 1997; Smith, 1956). Sonoran tiger salamanders are listed as endangered by the United States Fish and Wildlife Service (U.S. Fish and Wildlife Service, 1997). Collins (1981) and Collins et al. (1988) described life history variation in Sonoran tiger salamander and other subspecies of *A. mavortium* that occur in Arizona. Some larvae metamorphose at the end of their first season, becoming immature metamorphosed morphs, after which they grow outside of the aquatic habitat into a mature metamorphosed morph. Alternatively, the most common strategy is to remain in aquatic habitats and mature into paedomorphic gilled adults, or mature branchiate morphs that typically account for more than 95% of captures during the breeding season with many ponds approaching 100%. Mature metamorphosed salamanders return to ponds in late winter/early spring to breed (Collins et al., 1988). Historically, Sonoran tiger salamanders would have occurred primarily in ciénegas (spring‐fed wetlands) and possibly riverine marshes and backwaters. But starting in the 19th and early 20th centuries, with the destruction and alteration of these natural habitats, springs and wetlands in the San Rafael Valley have been replaced by stock tanks (Collins et al., 1988; Hendrickson & Minckley, 1985). As a consequence, while in the past Sonoran tiger salamanders would have relied on aquatic habitats, such as natural ponds, vernal pools, or lentic streams to breed, now they rely on stock tanks.

Decisions about which upland areas to protect for amphibian conservation can be informed by data on movement distances of local species away from aquatic habitat (Schonewald‐Cox, 1988; Semlitsch, 1998; Semlitsch & Bodie, 2003). Buffer zones have been suggested as a conservation tool if based on movement data to provide the appropriate scale (Semlitsch, 1998). Movement of six *Ambystoma* species in the eastern United States suggested a minimum of 164.3 m would be necessary to encompass the terrestrial needs for 95% of individuals (Semlitsch, 1998). Similarly buffer zones of 100–165 m (*Ambystoma maculatum*: Harper et al., 2008) and >630 m (*Ambystoma californiense*: Orloff, 2009; Trenham & Shaffer, 2005) were suggested for other salamanders. No studies have assessed the conservation impact of buffer zones; however, such an approach could be used to prioritize areas for preservation and target specific corridors for connectivity.

Most studies on *Ambystoma* terrestrial ecology have taken place in the eastern United States where species are found in mesic forests with more reliable breeding sites. Predictions from these studies may not apply to desert or grassland species that live where water availability is more dynamic and less predictable (Orloff, 2009; Searcy et al., 2013; Trenham & Shaffer, 2005). The terrestrial phase of the Sonoran tiger salamander life cycle is the only means of responding to drying or disease events, and thus is critical for population persistence and connectivity. However, the terrestrial ecology and demography of Sonoran tiger salamander is virtually unknown despite the major threats this species is facing such as its limited distribution, dependence on human‐constructed stock tanks, invasive species, and elevated risk of die‐offs due to disease or stock tank drying (U.S. Fish and Wildlife Service, 2002; Hossack et al., 2017). Genetic work suggests that Sonoran tiger salamander subpopulations may suffer from high inbreeding rates and reduced gene flow (Jones et al., 1988, 1995; Storfer et al., 2014). Previous genetic work in San Rafael Valley suggests evidence of a recent bottleneck possibly due to the introduction of predatory non‐native species, and amphibian epizootics. However, a third ecological reason for potential bottleneck is that this salamander now relies exclusively on human‐made tanks for breeding and human disturbance has generally made San Rafael Valley drier limiting gene flow among tanks, thus limiting genetic variability. Moreover, nearly all populations were in their genetic structure significantly subdivided from each other, indicating that, despite some inter‐tank distances are <1 km, salamanders are unlikely to move between ponds (Storfer et al., 2014).

Understanding the landscape of risk, movement and habitat use of mature metamorphosed salamanders in the face of human land use and climate change is necessary to develop effective landscape management actions to recover species (U.S. Fish and Wildlife Service, 2002). In this study, we assessed the movement ecology of Sonoran tiger salamanders in southern Arizona and survival probability after leaving the aquatic environment. Because of the summer monsoons, we expected a higher survival rate in the summer than spring because of a lower risk of desiccation and decreased levels of predation due to emergent green vegetation that accompanies the monsoons. With the arid conditions of the landscape, we expected Sonoran tiger salamanders to move farther than other *Ambystoma* species in search for food resources and water compared to those species that occur in the eastern United States where forested ephemeral wetlands provide reliable breeding and less arid upland habitat. We used radiotelemetry to follow movements from breeding ponds and monitor distances traveled and survivorship of animals leaving during the dry April–May spring season and the wet June–August periods to evaluate the consequences of movement.

## 2 MATERIALS AND METHODS

### 2.1 Study area

**FIGURE 1:**
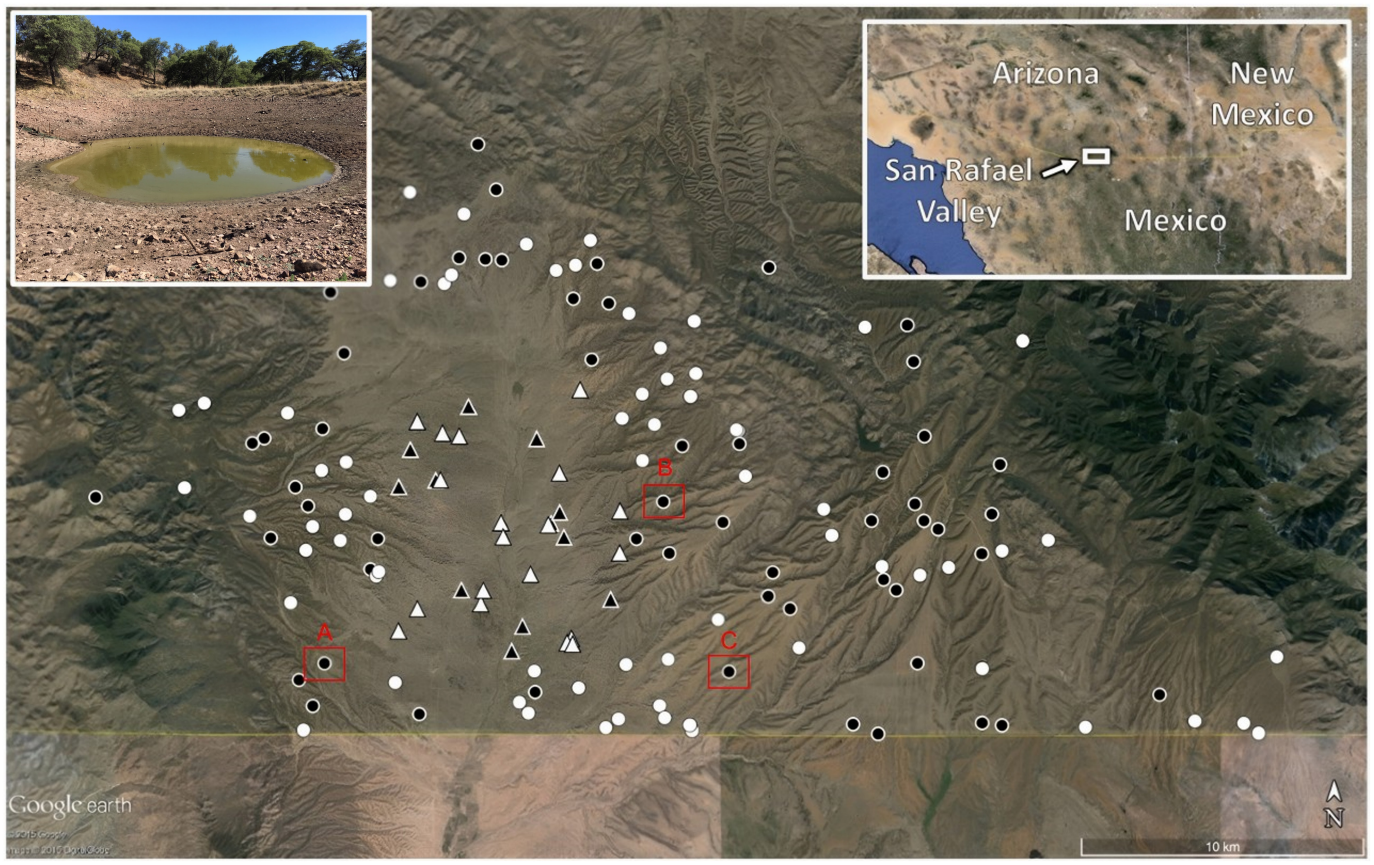
Stock tank locations in the San Rafael Valley, Santa Cruz County. Black shapes are tanks where salamanders were detected, and white shapes are tanks where salamanders were never detected from 2004 to 2013. Circles represent tanks that were part of the original sampling frame and triangles represent tanks on private land that were added to the monitoring program starting in 2009. The horizontal line is the USA‐Mexico border. Within the red squares the tanks object of this study: (a) Upper 13 Reservoir; (b) Dan Tank; (c) Huachuca Tank. Top left corner, Dan Tank. Figure 1 modified from Hossack et al., 2017.

The San Rafael Valley is an 86,000 ha grassland situated between the Patagonia and Huachuca mountains in southeastern Arizona and adjacent Sonora, Mexico (Hadley & Sheridan, 1995). The vegetation community is plains grassland transitioning into Madrean oak‐juniper woodland in surrounding foothills (Brown, 1994). Perennial grasses such as gramas (*Bouteloua* spp.), plains lovegrass (*Eragrostis intermedia*), and bluestems (*Andropogon* spp.) dominate the landscape (Brown, 1994; Hadley & Sheridan, 1995). Historically, in San Rafael Valley used to occur ciénegas (spring‐fed wetlands) and riverine marshes and backwaters. But starting in the 19th and early 20th centuries, with the destruction and alteration of these natural habitats, springs and wetlands in the valley have been replaced by stock tanks (Collins et al., 1988; Hendrickson & Minckley, 1985). These tanks are specifically designed to harvest runoff water for livestock use and are exceptional at capturing rainwater. Locations are carefully chosen to maximize this function. Usually, tanks are constructed using a bulldozer that scoops a shallow basin in the path of seasonal runoff water with an earthen dam created downslope. As water flows downslope, it collects in these earthen basins until reaching an average water depth of 1–1.5 m (Nichols & Degginger, 2021). Average nearest neighbor distance among all stock tanks in the valley is 760 ± 877 m. We focused our study at three stock tanks that typically have some water during all seasons (perennial tanks) in the San Rafael Valley with a history of relatively high presence of terrestrial morphs of Sonoran tiger salamander (>2%), Dan Tank, Upper 13 Reservoir, and Huachuca Tank (Collins et al., 1988) (Figure 1).

### 2.2 Trapping and handling

We captured mature metamorphosed individuals at the three stock tanks in March–August 2017 and March–July 2018. We caught salamanders by pulling a bottom‐weighed 9.1 m × 1.5 m bag seine across a tank (Hossack et al., 2017; Routman, 1984), and seined each tank one to ten times per sampling effort. Number of seine hauls varied based on capture rate, ending when a maximum of six mature metamorphosed salamanders were captured due to endangered status, or three consecutive hauls were empty. All equipment, clothing, and footwear was disinfected with quaternary ammonia (Quat‐128; 0.02% dilution) between sites to prevent disease transmission (Phillott et al., 2010). Individuals captured were weighed to nearest 1 g using a spring Pesola and only individuals weighing >38 g were transported to the Central Animal Facility at the University of Arizona for transmitter implantation. Juveniles, with their small body mass, were considered unfit for transmitter implantation. We also practiced the techniques on size non‐endangered invasive barred tiger salamanders (*A. m. mavortium*) from the Chiricahua Mountains, Cochise County, Arizona, and maintained these individuals in terraria for more than 1 year without any health‐related complications or issues. We anesthetized each metamorphosed salamander with a 0.2% solution of MS‐222 buffered to neutral pH, weighed them to the nearest 0.1 g with a digital scale (HL‐200i; A&D Co., Tokyo, Japan), identified sex, and used a ruler (±0.5 mm) to estimate snout‐vent length (SVL) and total length. This last measure was used to calculate salamander body condition via linear regression residuals of log‐transformed body mass on log‐transformed snout‐vent length (Jakob et al., 1996; Lowe et al., 2006; Schulte‐Hostedde et al., 2005). To assess movement and survival of mature metamorphosed morphs, we implanted a VHF radio transmitter (3.5 g, PD‐2HX, estimated battery life 7ths; Holohil Inc., Ontario, Canada) and a Passive Integrated Transponder (PIT) tag (9 mm, 65 ± 15 mg; Biomark Inc., Boise ID, USA) in each metamorphosed salamander (transmitter was <10% of total salamander mass). We conducted implantation surgeries in the Central Animal Facility, following Faccio (2003). We used an autoclave to sterilize surgery instruments before use. We made a small incision into the left ventrolateral wall 10 mm anterior to the hind leg and inserted the PIT tag and transmitter into the abdominal cavity. We closed the incision using dissolvable suture and applied analgesic and antiseptic spray (Bactine, WellSpring, Saratosa FL, USA). We held salamanders in the facility for 24 h before releasing them back into their capture site. All recovered radio implanted animals (alive or dead) were visually assessed for pathology and suture sites examined for healing. All field work was conducted under the University of Arizona Institutional Animal Care and Use Committee protocol #16‐147, Arizona Game and Fish Department scientific collecting permit #SP501610 for 2017 and #SP611944 for 2018, U.S. Fish and Wildlife Service permit #TE041875.

### 2.3 Movement

To determine Sonoran tiger salamander movements, we used digital receivers (R‐1000, Communication Specialists Inc., Orange, CA, USA) and yagi 3‐element directional antennae (Wildlife Materials Inc., Murphysboro IL, USA) to track implanted salamanders 2–4 times per week during the spring (March–May) and summer (June–August), and 1–2 times per week during the fall and winter (September–February) in 2017 and 2018. We determined locations by homing and recorded all locations on a handheld GPS unit (eTrex Legend Cx ± 5 m; Garmin, Olathe KS, USA). We also recorded the landscape feature where the salamander was located (e.g., burrow, rock, soil).

We used ArcGIS and Geospatial Modeling Environment software to calculate average nearest neighbor distance for known stock tank locations, based on prior Arizona Game and Fish Department surveys, to determine the dispersal distance necessary for Sonoran tiger salamander to reach a neighboring stock tank. We also calculated the sum of all distances between telemetry locations during tracking (hereafter, total traveled distance), the average distance traveled per day (hereafter, daily distance) and the linear distance between the stock tank edge and the farthest location recorded (hereafter, maximum distance). We used single linear regression models with Bonferroni correction to evaluate the relationship between movement metrics and salamander characteristics as predictors. Sex and body condition were tested to assess the effect of biological characteristics of the animal on movement, and tank of origin to account for possible differences in the landscape at our sampling sites. Year was not tested because we could estimate the home range of only two salamanders in 2018 (see Section 3). We used the same variables to model the average daily distance and the maximum distance.

To be able to compare this study with previous studies conducted on other *Ambystoma* species, we calculated home ranges using kernel density methods on ArcGIS v.10.6 and Geospatial Modeling Environment software (Beyer, 2014) and performed data analysis with the software R version 4.2.1 (R Development Core Team, 2021). The initial movement from the stock tank to a location that served as a terrestrial center of activity were excluded from home range analysis (Semlitsch, 1981). For salamanders with ≥20 locations, that is the minimum number of locations necessary to produce stable home range estimates, we used the fixed kernel density estimator (KDE) with maximum likelihood cross validation of the smoothing parameter (CVh) to estimate total home range (95% KDE). We modeled the relation between home range size and biological characteristics of the animal (sex and body condition) and tank of origin (to account for possible differences of the habitat at our three sampling locations) fitting separate linear regressions with Bonferroni adjustment of p‐values (the small sample size did not allow for a multiple regression analysis). Again, year was not tested because we could estimate the home range of only two salamanders in 2018 (see Section 3). Home range values were square root‐transformed to meet assumption of normality. We expected that movement during the dry spring season would result in low survivorship due to desiccation and low cover from dried vegetation compared to the wet seasons characterized by summer monsoons with a flush of plant cover that follows.

### 2.4 Survival

We used the R package ‘survival’ (Therneau et al., 2022; Therneau & Grambsch, 2010) to assess the survival probability of salamanders after leaving the tank (overall and stratified by season) with a Kaplan–Meier survival curve (Kaplan & Meier, 1958; Pollock et al., 1989). We also fitted a Cox Proportional Hazards Model (Cox, 1972) to assess the effect of body condition, season (April + May = spring; June + July = summer) and tank of origin on the probability of survival.

All mean values are expressed as ± the standard deviation (SD), unless stated otherwise.

## 3 RESULTS

### 3.1 Stock tank surveys and salamander measurements

During 45 stock tank surveys over 38 total survey days (average 3.51 ± 2.27 seine pulls/survey), we captured 172 mature metamorphosed morphs, 2949 mature branchiate morphs, and 7231 larval and juvenile salamanders (Table 1). The skewed ratio of 0.058:1 terrestrial: branchiate mature morphs captured demonstrates the relative rarity of terrestrial morphs in this system. Male to female sex ratio for all mature salamander captured was 1.42:1. We captured and implanted 78 salamanders, 38 in 2017 and 40 in 2018 (40 males and 38 females). The implanted salamanders were those whose body weight was large enough to carry transmitters and were selected to be comparable in number per tank and sex. Implanted males had a mean body mass of 52.07 ± 10.02 g and a SVL of 12.22 ± 0.54 cm; females on average had a body mass of 63.63 ± 14.21 g and a SVL of 12.19 ± 0.69 cm. We sampled Dan Tank 17 times, Huachuca Tank seven times, and Upper 13 Reservoir 18 times. Midway through 2018 Huachuca Tank water level dropped and the remaining water was too rich in sediment to continue sampling thus resulting in a lower number of sampling opportunities.

**TABLE 1:** Sonoran tiger salamanders (*Ambystoma mavortium stebbinsi*) captured in 2017 and 2018 from three surveyed stock tanks in the San Rafael Valley, Arizona.

Site	Year						Total		
	2017			2018					
	Meta	Branch	Juv	Meta	Branch	Juv	Meta	Branch	Juv
Dan tank	9	1044	708	14	752	890	23	1796	1598
Huachuca tank	11	83	1324	2	168	314	13	251	1638
Upper 13 reservoir	18	165	2630	24	737	1365	42	902	3995
Total	38	1292	4662	40	1657	2569	78	2949	7231

*Note*: “Meta” = Metamorphosed adults captured and implanted; “Branch” = mature branchiate morphs captured; “Juv” = juvenile and larval individuals captured.

### 3.2 Movement

Using the technique of homing, we determined that none of the implanted salamanders dispersed to a different stock tank (any stock tank on the landscape) during their tracking period. On average, implanted salamanders that left the tank (*n* = 67) remained in the tank for 21 ± 13.9 days (range 4–61) after transmitter implantation before moving out of the tank. Upon leaving the tank, average time between salamander movement events was 7.6 ± 6.6 days (range 1 to 39 days).

**FIGURE 2:**
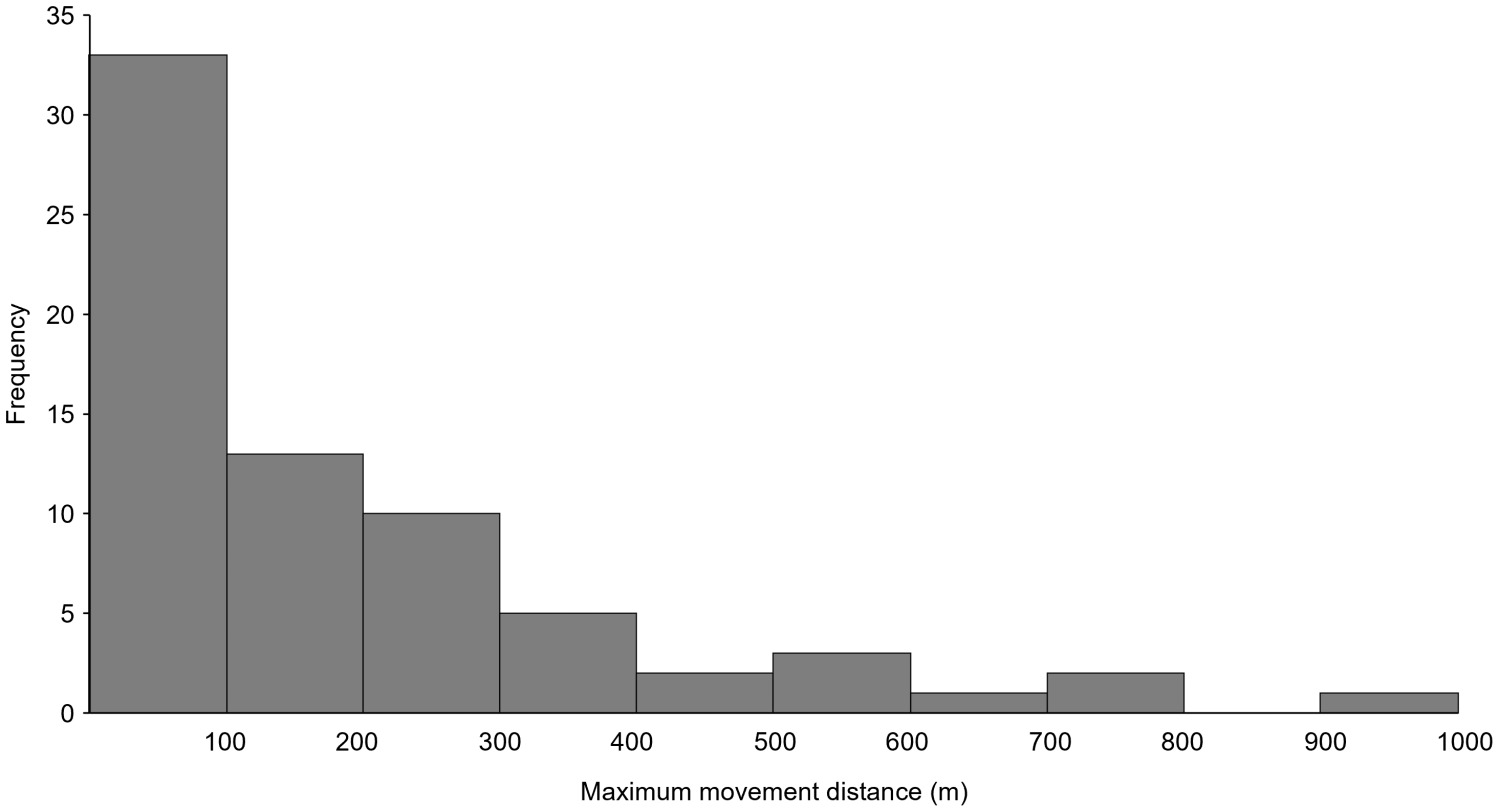
Maximum movement distance from stock tank for radio‐transmitter implanted Sonoran tiger salamanders (*Ambystoma mavortium stebbinsi*) in the San Rafael Valley, Arizona (*n* = 77).

We analyzed total and daily traveled distance and maximum distance from tank edge for implanted salamanders with at least 20 locations (*n* = 11). We also recorded the maximum distance traveled from the tank edge for implanted salamander (*n* = 77, Figure 2). Average tracking time was 190 ± 92 days (range 77–361). Mean total traveled distance was 751.5 ± 353.8 m, mean maximum distance moved was 403.6 ± 215.2 m and mean daily distance was 4.52 ± 2.30 m. One salamander that was tracked for a short period (nine locations) before going missing, traveled a maximum distance of 974 m from tank edge. None of the variables tested (sex, body condition, tank) explained the average daily distance or the maximum distance traveled by salamanders (all *p* > .02).

We were able to calculate the home range of only 11 salamanders (6 M, 5 F) with ≥20 locations (see Supporting Information). We found a high individual variability in home range size (mean home range: 3054 ± 4301 m^2^) and it was not explained by body condition, tank of origin or sex (all *p* > .02). Previous work on other *Ambystoma* species showed that metamorphosed adults spend much of the year in subterranean terrestrial habitat before returning to their natal breeding site (Gamble et al., 2007; Semlitsch, 1981; Trenham et al., 2000; Whiteman, 1994). In fact, salamanders in San Rafael Valley were located most often within small mammal burrows (96% of salamander locations in small mammal burrows, 2% leaf litter, 2% tree stumps, <1% rock ledges).

### 3.3 Survival

Of the 78 salamanders captured, we analyzed terrestrial survival on a subset of 58 salamanders (M = 29, F = 29), 17 that dispersed in spring and 41 in summer. Eleven were excluded because they never dispersed from the tank (three confirmed mortalities and for other eight transmitter battery expired). Nine subterranean individuals were considered mortalities based on a complete halt of all movement long before transmitter battery expiration (≥77 days). These subterranean individuals were excluded from survival analysis because the exact date and cause of death was unknown. A University of Arizona veterinarian performed a necropsy on one of the confirmed tank mortalities and concluded that transmitter implantation was not the cause of death; in addition, field teams assessed the health of 14 live and 12 dead implanted animals in the field without any pathology or complications at suture sites as noted in other implantation studies (Faccio, 2003; Madison, 1997).

**FIGURE 3:**
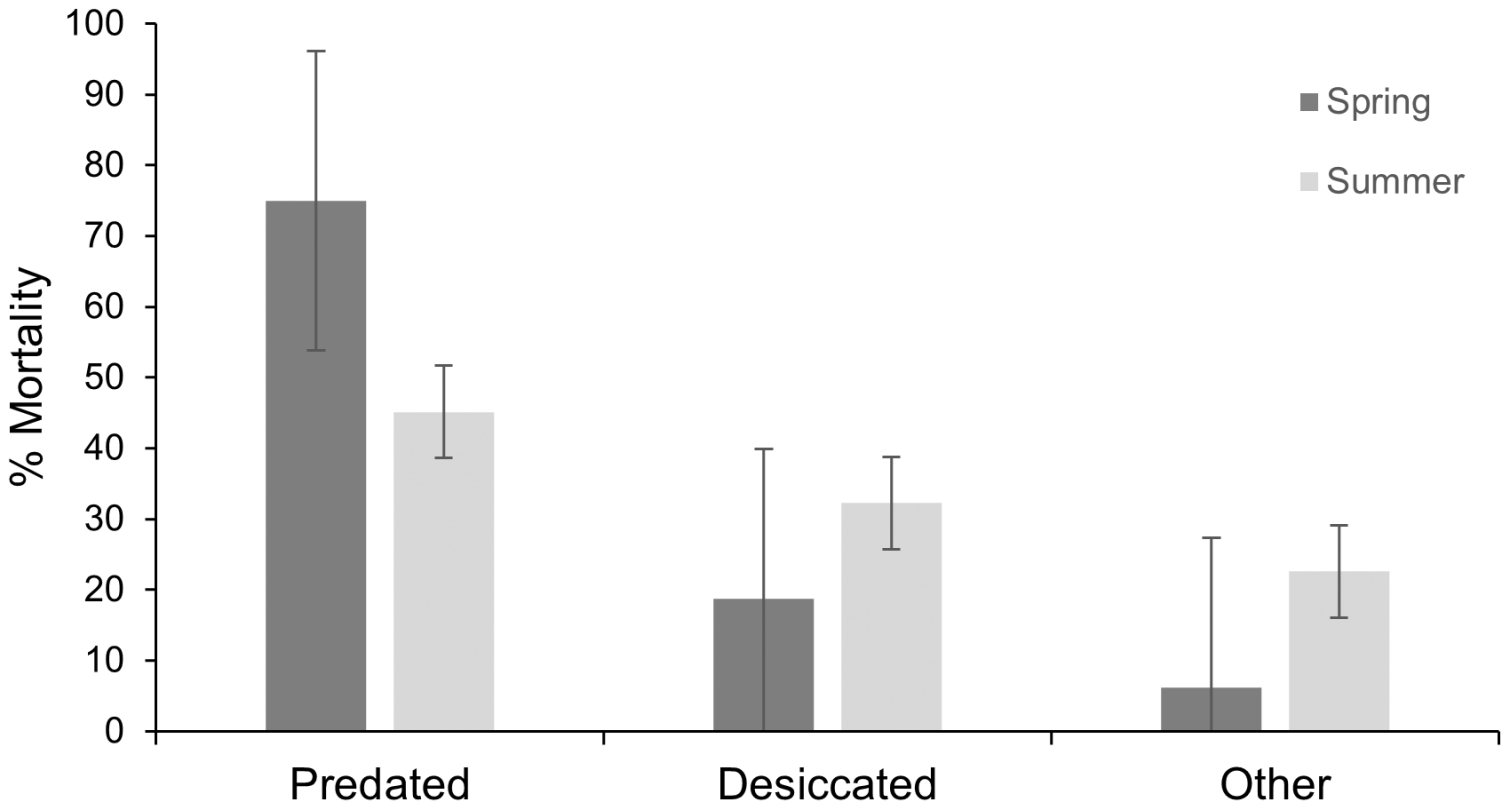
Causes of mortality following dispersal for radio‐transmitter implanted Sonoran tiger salamanders (*Ambystoma mavortium stebbinsi*) in the San Rafael Valley, Arizona (*n* = 58). Percentage of mortalities and error bars. Spring season (April–May) dispersers in dark gray and summer dispersers (June–July) in light gray.

**FIGURE 4:**
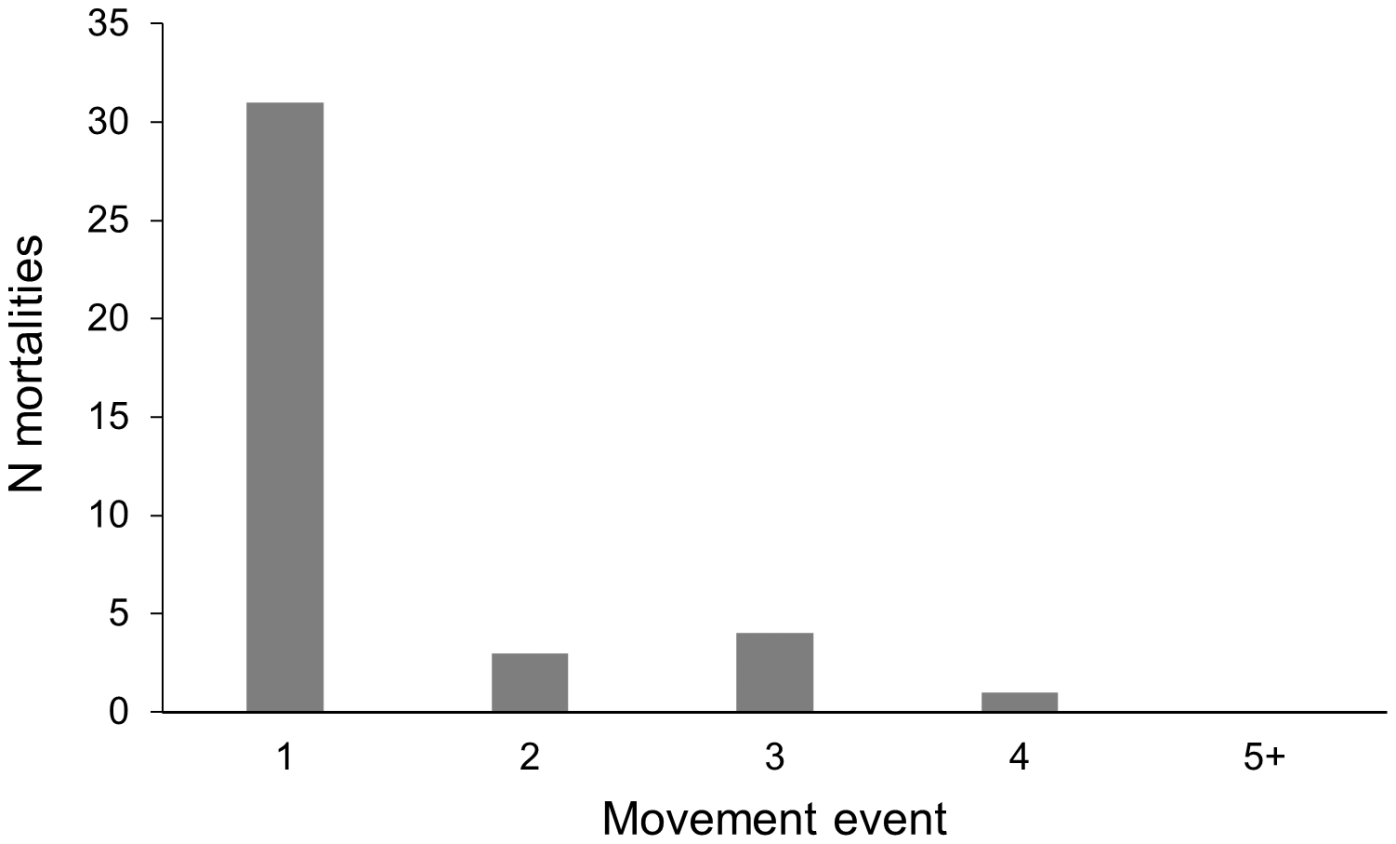
Number of mortalities for radio‐transmitter implanted Sonoran tiger salamanders (*Ambystoma mavortium stebbinsi*) in the San Rafael Valley, Arizona according to the documented movement number when they occurred. Movement 1 is initial movement out of tank, movement 2 is second movement event, and so forth.

We recorded high mortality rates during our study. Of the 58 individuals in the analysis, only 11 salamanders survived (81.03% mortality) until transmitter expiration (*X* = 190 ± 91.9 days; range 77–361 days). The most common cause of terrestrial mortality was predation (*n* = 26; 44.8%; Figure 3) and it was identified by the discovery of a transmitter, often with a yellow‐green residue from gut contents, and occasionally indications of tooth or beak marks. Some transmitters from depredated salamanders were broken, and one was recovered from an owl pellet. The second most common cause of terrestrial mortality was desiccation (*n* = 13; 22.4%). Desiccated salamanders were found dried on the ground with no evidence of predation. Despite thick grass‐cover, we observed three desiccated salamanders that were not tagged and included in the study near our study sites, revealing that desiccation may be common in the San Rafael Valley. Mortality was most common following the first documented movement out of tank, and all predation or desiccation events occurred between the first and fourth documented movement event (Figure 4). All individuals that survived past their fourth movement event, survived until the end the tracking period. Of the 47 salamanders that died (M = 24, F = 23), individuals spent an average of 4.6 ± 9.6 terrestrial days (range 1–41) before mortality.

**FIGURE 5:**
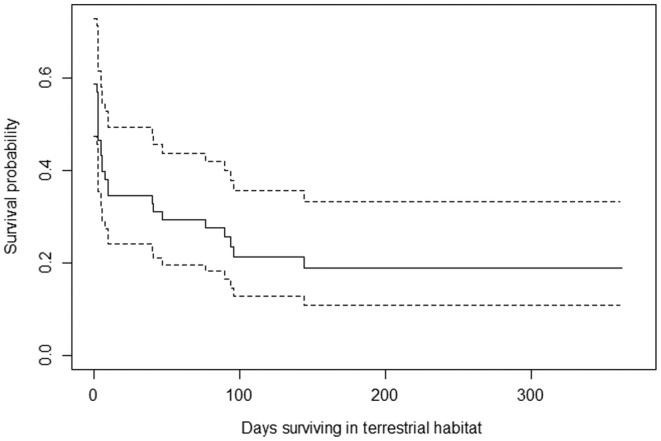
Survival curves for radio‐transmitter implanted Sonoran tiger salamanders (*Ambystoma mavortium stebbinsi*) in the San Rafael Valley, Arizona after leaving stock tank. Overall survival probability (solid line) and 95% CI (dashed line).

Overall, terrestrial Sonoran tiger salamanders that survived past their fourth movement event survived for 82.5 days ± SE 20.9. Upon departure from the tank, salamanders had a survival probability of 0.60 (95% CI 0.47–0.73) that gradually decreased over time to stabilize at 0.02 after 144 days out of the tank (95% CI 0.11–0.33) (Figure 5). Individuals that dispersed to terrestrial habitat in spring (*n* = 17; *X* = 21.8 ± SD 14.9 days) survived less than individuals that dispersed in summer (*n* = 41) (*X* = 73.9 ± SD 19.1 days) (−0.70 ± SE 0.50, *z* = −2.05, *p* = .04). Of the individuals that did not survive in the spring, 75% were predated. Of the individuals that did not survive in the summer, 45% were predated (Figure 3). Survival probability was similar for salamanders originated in the three different tanks and body condition was not influential (all *p* > .05).

## 4 DISCUSSION

As revealed by intensive radiotelemetry of implanted rare and secretive salamanders, terrestrial life comes at a high price in a hydrologically fragmented landscape in arid lands, with high mortality due to depredation and desiccation, risk that seems to be attenuated in the summer. The scarcity of terrestrial morphs (<6%) of all adult animals in our fishless breeding ponds is likely a reflection of the cost of terrestrial movement via low survivorship.

### 4.1 Movements suggest low connectivity

Terrestrial movements paint a picture of a matrix between aquatic habitats that is quite hostile in arid lands with periods of spatial stasis punctuated by rapid movement. During the tracking period, Sonoran tiger salamanders moved an average of 751.5 m, 0.7–6.7 times farther from aquatic habitat than distances reported for other *Ambystoma* species (Table 2). These prior studies of *Ambystoma* were conducted in the eastern United States where water and forest cover are more common. Reduced water availability in the semiarid grasslands of the San Rafael Valley may contribute to further Sonoran tiger salamander movement in search for water and moist microclimates that are increasingly rare across the landscape. *A. californiense* in the western United States moved a median distance of 556 m (Searcy et al., 2013), suggesting ecological similarity to Sonoran tiger salamanders. Buffer zone suggestions based on these eastern *Ambystoma* movements are not sufficient to encompass Sonoran tiger salamander movement distances. Based on Semlitsch's use of the 95th percentile of movement distances to estimate buffer zone extent (Semlitsch, 1998), we suggest a buffer zone of 800 m for conservation management of these populations to ensure adequate connectivity between breeding locations. Our results are consistent with those of Searcy et al. (2013) who recognized that management recommendations for eastern *Ambystoma* species cannot be applied to western species. Terrestrial habitat protection for amphibians should be based on site‐specific understanding of amphibian movement (Dodd Jr. & Cade, 1998; Regosin et al., 2005; Titus et al., 2014). Terrestrial home range size for Sonoran tiger salamanders was larger than that recorded for other *Ambystoma* species (Table 3). Although different home range estimators with small samples sizes can be problematic, the home range of Sonoran tiger salamander was two orders of magnitude larger. We found no relation between our measures of body condition and home range size or distance traveled; however, our sample sizes were small. The few studies about the movement ecology of *Ambystoma* species (Tables 2 and 3) had a small sample size to quantitatively assess differences between males and females (Semlitsch, 1981) or did not find any significant difference between sexes (Kleeberger & Werner, 1982; Madison & Farrand, 1998; Ousterhout & Burkhart, 2017). Space use during the terrestrial life stage of this species might depend more on ecological factors such as habitat quality or predator density than on individual traits, as it was also suggested for *A. annulatum* and *A. maculatum* (Ousterhout & Burkhart, 2017).

**TABLE 2:** Mean distance traveled by salamanders of the genus *Ambystoma.*

Species	Distance (m)	Location	Reference
*Ambystoma tigrinum*	60.5	New York, USA	Madison and Farrand (1998)
*Ambystoma tigrinum*	110.7	Georgia, USA	Steen et al. (2006)
*Ambystoma maculatum*	118	Michigan, USA	Kleeberger and Werner (1982)
*Ambystoma talpoideum*	178	South Carolina, USA	Semlitsch (1981)
*Ambystoma maculatum*	192	New York, USA	Madison (1997)
*Ambystoma opacum*	193.7	Indiana, USA	Williams (1973)
*Ambystoma jeffersonianum*	252	Indiana, USA	Williams (1973)
*Ambystoma opacum*	297^a^	Massachusetts, USA	Gamble et al. (2007)
*Ambystoma californiense*	556	California, USA	Searcy et al. (2013)
*Ambystoma mavortium stebbinsi*	751.5	Arizona, USA	This study

^a^ Mode.

**TABLE 3:** Mean home range size of salamanders of the genus *Ambystoma.*

Species	Home range (m^2^)	Estimator	Location	Reference
*Ambystoma talpoideum*	M: 3.61; F: 5.29	MCP	South Carolina, USA	Semlitsch, 1981
*Ambystoma maculatum*	9.83	MCP	Michigan, USA	Kleeberger & Werner, 1982
*Ambystoma annulatum*	28.68	KDE	Missouri, USA	Ousterhout & Burkhart, 2017
*Ambystoma maculatum*	41.43	KDE	Missouri, USA	Ousterhout & Burkhart, 2017
*Ambystoma mavortium stebbinsi*	3054	KDE	Arizona, USA	This study

Despite the ability for long‐distance movement, none of our telemetered salamanders reached a different stock tank during the time they were tracked, and on average did not move as far as the average nearest distance for neighboring stock tanks in the valley. However, we were not able to measure juvenile dispersal as their small body mass rendered them unfit for transmitter implantation. This may have reduced movement estimates, as juveniles are known to be a primary life stage for dispersal in other amphibians (Cayuela et al., 2020; Cushman, 2006; Gill, 1978; Trenham & Shaffer, 2005). High mortality of terrestrial salamanders suggests that movements in the landscape are constrained by the high risk of predation and desiccation. With connectivity constrained, it is likely that Sonoran tiger salamander movements may not provide continuous connectivity between subpopulations and a very small number of successful long‐distance dispersers account for colonization of stock tanks after local extinction events and provide gene flow to isolated subpopulations (Mills & Allendorf, 1996). Given the small proportion of mature metamorphosed salamanders in this site, the high mortality and likely rare movement to other aquatic environments of these adults is unique for the genus. Thus the question that arises is whether this terrestrial stage in an arid environment, such as the Sonoran Desert, is an evolutionary vestige of the salamander's temperate forest origins. In this area, a climatic shift in recent decades or centuries may have severely affected the ecological success of different life stages that in the past experienced a higher terrestrial success as did their eastern counterparts. However, despite the successful movement to new stock tanks is rare, it could be functional to metapopulation dynamics. In addition to successful movements into new stock tank, random events such as flash‐floods associated with seasonal monsoons might cause passive movement facilitating as well gene flow among subpopulations (Adams & Comrie, 1997).

### 4.2 Movements in a semiarid landscape are risky

Arid lands represent a coarse‐grained matrix that is abrupt and harsh to travel for many species, especially amphibians. *Ambystoma* species are vulnerable to high rates of desiccation in unsuitable conditions, especially if sufficient refuge is not available (Holland et al., 1990; Rothermel & Luhring, 2005; Semlitsch, 1983). Increased aridity may also cause moisture‐dependent tradeoff between the production of alarm chemical cues in amphibians and anti‐predator behavior, thus increasing predation risk (Rohr & Madison, 2003). The survival rates that we observed document the risky nature of the post‐breeding or post‐metamorphic dispersal on the breeding ponds. Our opportunistic assessment of full apparent recovery from surgery as in other radiotelemetric studies (Faccio, 2003), general timing of movements with patterns of rain and humidity, and fate of known mortalities suggest that our data provide an effective assessment of success of terrestrial life of metamorphosed adults. The survival rate recorded in this study is not directly comparable to estimates of previous studies on *Ambystoma* species. Annual mortality for *A. maculatum* (Whitford & Vinegar, 1966), *A. talpoideum* (Raymond & Hardy, 1990), and *A. californiense* (Trenham et al., 2000) were 10.5%, 25.8% and 50%, respectively. However, the estimates in this study, despite being higher, are related just to the terrestrial stage of Sonoran tiger salamanders that is not representative of the entire breeding population. Longitudinal studies across multiple years can provide greater insight into the risks of multiple movements across a hostile landscape and the true fitness cost experienced by terrestrial individuals that could be important for a rescue effect in a metapopulation framework.

Mature metamorphosed morphs had a higher survival percentage when dispersing in the summer monsoon. Studies of other *Ambystoma* species suggest that breeding migrations often occur during rain events (Semlitsch, 1981; Trenham et al., 2000). Southern Arizona exhibits a bimodal rainfall pattern, with winter rains from December through February and heavy summer monsoon rains from late June to August (Adams & Comrie, 1997; Smith, 1956). Desiccation and depredation were more likely in the spring, possibly due to the lack of vegetative cover before monsoon rains that causes a lack refuge sites and a higher predation risk that has been shown to increase near breeding ponds during times of amphibian breeding migrations (Rittenhouse et al., 2009). We do not have data on predator pressure during these different seasons; however, the vast majority of predators are active and present in the region in both spring and summer. Changes in the climate regimes of the region could worsen or might already be reducing the chances of survival of the terrestrial life stage of Sonoran tiger salamander; this, paired with the high mortality recorded resulting from risky terrestrial movement, may be contributing to the loss of genetic connectivity among subpopulations (Storfer et al., 2014).

Sonoran tiger salamander larvae either metamorphose or exhibit paedomorphosis to become mature branchiate morphs. Paedomorphosis is selectively maintained by different environmentally variable fitness payoffs to each morphology (Routman, 1993; Whiteman, 1994; Whiteman et al., 1996; Wilbur & Collins, 1973). One mechanism, the “Paedomorph Advantage,” suggests that paedomorphs have a fitness advantage by remaining in aquatic habitat, and avoiding extreme terrestrial conditions (Whiteman, 1994). High terrestrial mortality risk resulting from desiccation or depredation in the San Rafael Valley, coupled with increased water permanence of stock tanks, likely select against dispersing salamanders. This hypothesis seems supported by the high percentage of mature branchiate morphs captured compared to mature metamorphosed morphs that might reflect that selection is favoring the paedomorphic compared to the metamorphic life history. Persistence of Sonoran tiger salamander populations might be demographically dependent from the paedomorph life history, while the metamorphosed morphs are only important for occasional gene flow and so the success of such dispersal would only require favorable moisture conditions on rare occasions.

### 4.3 Management of landscapes for connectivity is key

Management to improve connectivity is a major challenge in conservation and of particular importance in these arid working lands (Mims et al., 2015). In particular, protection of isolated aquatic environments such as stock tank habitats is essential as exemplified by their importance to the recovery of this endangered species. Potential management actions such as the removal of invasive predators [i.e., non‐native fish, bullfrogs, and crayfish, (Hossack et al., 2017)], creation of stepping‐stone aquatic habitats, and translocation of individuals between stock tanks may improve population persistence and gene flow (Hale et al., 2015; Semlitsch, 2002; Snow & Witmer, 2010; Weeks et al., 2011). However, these actions are labor intensive and may have unintended consequences such as increased encroachment by invasive predators via stepping‐stone habitats (Hossack et al., 2017; McIntyre et al., 2016). Because many invasive predators such as fish, frogs, and crayfish can require permanent water to persist, the ability of some Sonoran tiger salamanders to leave the ponds can perhaps provide an opportunity for persistence during extreme droughts and even provide short periods to target removal of or treatment for invasives. Studies to preserve buffer zones and enhance small‐scale corridors for movement should be pursued as these areas become critical for persistence and likely can decrease predation and desiccation, which are the primary sources of mortality. Our results suggest that modest buffer zones would likely be impactful for migrating salamanders and should be explored. Given the poor survivorship of terrestrial morphs and pending studies on the effectiveness of buffer zone habitat management, a small number of human‐assisted translocations between stock tanks could be given consideration to maintain gene flow where that is a concern. Furthermore, for amphibian conservation efforts to be successful, partnership between landowners, natural resource managers, and other stakeholders is critical (Rosen & Schwalbe, 1998), and the stakeholders involved in the Sonoran tiger salamander conservation and management in the San Rafael Valley must continue their collaborative efforts to maintain aquatic habitats and implement conservation strategies for this unique and imperiled amphibian. Such actions provide a path forward in heavily fragmented environments where the cost of dispersal is exceptionally high, yet the process of dispersal is required to prevent extinction or to promote genetic and demographic rescue. Understanding animal movement is crucial for managing and conserving functional ecosystems in the Anthropocene. This is especially important in environments that are heavily impacted by human activities and affected by climate change. Such habitats are often fragmented and have harsh conditions, making it necessary to monitor and track animal movement to ensure their survival (Baguette et al., 2012; Cote et al., 2017).

## AUTHOR CONTRIBUTIONS

**Colin W. Brocka:** Conceptualization (equal); data curation (equal); formal analysis (supporting); funding acquisition (equal); investigation (lead); writing – original draft (equal). **Maria Vittoria Mazzamuto:** Formal analysis (lead); methodology (equal); validation (equal); visualization (equal); writing – original draft (equal); writing – review and editing (equal). **John L. Koprowski:** Conceptualization (equal); funding acquisition (lead); methodology (equal); project administration (lead); resources (equal); supervision (equal); validation (equal); writing – review and editing (equal).

## CONFLICT OF INTEREST STATEMENT

No conflict of interest.

## Supporting information


Data S1

## Data Availability

Data is available at Dryad doi: https://doi.org/10.5061/dryad.p2ngf1vvg.
