# Supplementary material for: The high cost of movement in an arid working landscape for an endangered amphibian
Source: Ecol Evol. 2024 Apr 16;14(4):e11259. doi: 10.1002/ece3.11259 (PMC11019298; doi:10.1002/ece3.11259)
Supplement: Supplementary file 1 — Data S1 [file ECE3-14-e11259-s001.docx]

*Home Range*

We were not able to calculate home range and core area size of 67 salamanders that were implanted: 11 never left the tank, 1 left the tank for a day then returned where it died 94 days after implantation, 9 went missing or the transmitter failed within a few days of their release, 7 left the tank but did not move after settlement and remained underground, and 39 were depredated or desiccated within 10 days from implantation (see paragraph Survival for more details). We calculated the home range and core area size of the remaining 11 salamanders (6 M, 5 F) with ≥ 20 locations. We collected 647 total telemetry locations of terrestrial salamanders with a mean number of fixes per individual of 39 ± 13 (range 20-65). Mean terrestrial home range size was 3,054 $\pm$ 4,301 m^2^, and the core area was 505 $\pm$ 610 m^2^.

| **ID** | **Tank** | **Sex** | **Mass** | **SVL** | **Total_L** | **Tail_H** | **Home range** | **Core area** |
| --- | --- | --- | --- | --- | --- | --- | --- | --- |
| D5 | Dan | F | 59 | 12 | 25.1 | 1.3 | 15086.47 | 2102.80 |
| D6 | Dan | M | 46 | 12.6 | 22.7 | 1.7 | 3007.25 | 690.74 |
| D27 | Dan | M | 59.6 | 12.7 | 23.7 | 1.6 | 222.27 | 35.03 |
| H3 | Huachuca | F | 63 | 12.1 | 22.4 | 1.8 | 716.23 | 90.80 |
| H6 | Huachuca | F | 79.1 | 12.7 | 23.7 | 2.2 | 861.70 | 158.80 |
| H9 | Huachuca | F | 64.6 | 12.4 | 22.6 | 2 | 7392.76 | 1129.32 |
| U5 | Upper 13 | M | 41 | 11.4 | 22.5 | 1.9 | 46.90 | 0.25 |
| U8 | Upper 13 | M | 42 | 11.6 | 23.2 | 1.9 | 955.11 | 129.445 |
| U12 | Upper 13 | M | 60.5 | 12.5 | 23.5 | 1.7 | 2678.15 | 648.03 |
| U18 | Upper 13 | M | 59.6 | 12 | 22.5 | 2 | 327.06 | 70.32 |
| U35 | Upper 13 | F | 42.8 | 10.7 | 18.1 | 2.2 | 2296.73 | 498.99 |

On average, females had a home range of 5,271 ± 6,121.26 m^2^, core area 796.15 ± 838.21 m^2^; males had a home range of 1,206 ± 1,308.31 m^2^, core area 262.31 ± 318.49 m^2^.
